# Association between the metabolome and bone mineral density in a Chinese population

**DOI:** 10.1016/j.ebiom.2020.103111

**Published:** 2020-11-10

**Authors:** Zhendong Mei, Xin Dong, Yu Qian, Dun Hong, Ziang Xie, Guanfeng Yao, An Qin, Songyan Gao, Jianying Hu, Liming Liang, Yan Zheng, Jiacan Su

**Affiliations:** 1 State Key Laboratory of Genetic Engineering, Human Phenome Institute, and School of Life Sciences, Fudan University, Shanghai, China; 2 Institute of translational medicine, Shanghai University, Shanghai, China; 3 School of Medicine, Shanghai University, Shanghai, China; 4 Department of Orthopaedics, Shaoxing People's Hospital (Shaoxing Hospital, Zhejiang University School of Medicine), Shaoxing, China; 5 Orthopedic Department, Taizhou Hospital Affiliated to Wenzhou Medical University, Linhai, China; 6 Department of Orthopaedics, Sir Run Run Shaw Hospital, Zhejiang University School of Medicine, Hangzhou, China; 7 Key Laboratory of Musculoskeletal System Degeneration and Regeneration Translational Research of Zhejiang Province, Hangzhou, China; 8 The Department of Orthopedics, Second Affiliated Hospital of Shantou University Medical College; 9 Department of Orthopaedics, Shanghai Key Laboratory of Orthopaedic Implant, Shanghai Ninth People's Hospital, Shanghai Jiaotong University School of Medicine, Shanghai, China; 10 Departments of Epidemiology and Biostatistics, Harvard T.H. Chan School of Public Health, 02115, Boston, MA, USA; 11 Ministry of Education Key Laboratory of Public Health Safety, School of Public Health, Fudan University, Shanghai, China; 12 Department of Orthopedics Trauma, Shanghai Changhai Hospital, Naval Medical University, Yangpu District, Shanghai, China

**Keywords:** Bone mineral density, Osteoporosis, Biomarkers, Metabolomic profiling

## Abstract

**Background:**

Osteoporosis is a common metabolic bone disease, which always leads to osteoporotic fractures. Biomarkers of bone mineral density (BMD) are helpful for prevention and early diagnosis of osteoporosis. This study aims to identify metabolomic biomarkers of low BMD.

**Methods:**

We included 701 participants who had BMD measures by dual-energy X-ray absorptiometry scans and donated fasting plasma samples from three clinical centres as a discovery set and another 278 participants from the fourth centre as an independent replication set. We used a liquid chromatography-mass spectrometry-based metabolomics approach to profile the global metabolites of fasting plasma.

**Findings:**

Among the 265 named metabolites identified in our study, six were associated with low BMD (FDR-adjusted *P*<0.05) in the discovery set and were successfully validated in the independent replication set. The circulating levels of five metabolites, i.e., inosine, hypoxanthine, PC (O-18:0/22:6), SM (d18:1/21:0) and isoleucyl-proline were associated with decreased odds of low BMD, and PC (16:0/18:3) level was associated with increased odds of low BMD. Per 1-SD increase in a composite metabolite score of these six metabolites was associated with about half decreased odds of low BMD (odds ratio 0.59, 95% confidence interval: 0.52-0.68). Furthermore, introduction of a panel of metabolites selected by elastic net regression to a prediction model of classical risk factors and plasma biomarker of bone resorption substantially improved the prediction performance for low BMD (AUCs: 0.782 vs. 0.698, *P*=0.002).

**Interpretation:**

Metabolomics profiling may help identify novel biomarkers of low BMD and be helpful for early diagnosis of osteoporosis beyond the current clinical index.

**Funding:**

This study was supported by the National Key R&D Program of China [2018YFC2001500 to J.S.], Shanghai Municipal Science and Technology Major Project [2017SHZDZX01], the National Natural Science Foundation of China [Key Program, 91749204 to J.S.], the National Natural Science Foundation of China [General Program, 81771491 to J.S.], the Project of Shanghai Subject Chief Scientist [2017BR011 to J.S.], Grants from the TCM Supported Project [18431902300 to J.S.] from the Science and Technology Commission of Shanghai Municipality, and the National Natural Science Foundation of China [General Program, 81972089 to Z.X.]. Y.Z. was supported by the Program for Professor of Special Appointment (Eastern Scholar) at Shanghai Institutions of Higher Learning, and the National Natural Science Foundation of China [81973032].

**Research in context**

**Evidence before this study**

Biomarkers of bone mineral density (BMD) are helpful for prevention and early diagnosis of osteoporosis. Large-scale metabolomics studies may provide novel predictive biomarkers.

**Added value of this study**

Using a comprehensive liquid chromatography-mass spectrometry-based metabolomics profiling approach, we examined the associations of circulating metabolites with low BMD in a large Chinese population, and replicated such associations in an independent population. We found several plasma metabolites were associated with low BMD, and a panel of selected metabolites significantly improved the performance of the model of classical risk factors and a bone resorption marker at distinguishing low BMD group from normal BMD group.

**Implications of all the available evidence**

Our results, taken together with prior evidence, highlighted that circulating metabolites may provide novel biomarkers for the early diagnosis of low BMD.

Alt-text: Unlabelled box

## 1 Introduction

Osteoporosis, a chronic condition characterized by a decrease in bone mineral density (BMD), structural deterioration of microarchitecture, and skeletal fragility,[1] is emerging as a global epidemic as people have a longer lifespan. More than 200 million people globally are suffering from osteoporosis.[2] Low BMD was a strong risk factor for fracture, and each lower standard deviation (SD) in BMD was associated with a doubled risk in fracture.[3] Among 1 in 3 women and 1 in 5 men who are over 50 years of age will experience osteoporotic fractures,[2] which usually results in a dependent living situation and premature death.[3] Low BMD and osteoporosis are silence, and the benefit-to-risk ratio for treatment is highly promising.[3] Therefore, BMD measurement was recommended at or around 65 years of age.[3] The current gold standard for BMD measure is the dual-energy X-ray absorptiometry (DXA) scan. However, a more comprehensive assessment of clinical risk factors and novel biomarkers is in need to evaluate risk and early prevention.[3]

Circulating biomarkers are clinically used to reflect different processes of bone metabolism. For example, C-terminal telopeptide of type I collagen (CTX-I) indicates the resorption of old bone, and N-terminal propeptide of type I procollagen (PINP) indicates the formation of new bone,[4] and they could be used to monitor bone response to therapy.[5,6] However, these available biomarkers are unreliable and do not improve prediction of bone loss or fracture, and thus are not recommended for diagnosis.[6] The systematic metabolic state of the human body is a determinant of skeletal health, and the identification of novel circulating biomarkers is urgently needed for early diagnosis of osteoporosis and prevention of osteoporotic fractures. Metabolomics has provided the potential to discover novel biomarkers for common metabolic diseases, e.g., diabetes and cardiovascular disease.[7,8] Nevertheless, large-scale metabolomics studies are highly needed to provide reliable and predictive biomarkers of osteoporosis.

Here, by utilizing a comprehensive liquid chromatography-mass spectrometry (LC-MS) based metabolomics profiling approach, we aimed to examine the association of plasma metabolites with BMD and osteoporosis among 701 Chinese participants and replicate our findings in an independent group of 278 participants. Furthermore, we assessed the predictive ability of the selected metabolites for bone loss and examined the extent of enhanced predictive ability from these metabolites beyond conventional clinical factors.

## 2 Methods

### 2.1 Study design and population

**Fig. 1:**
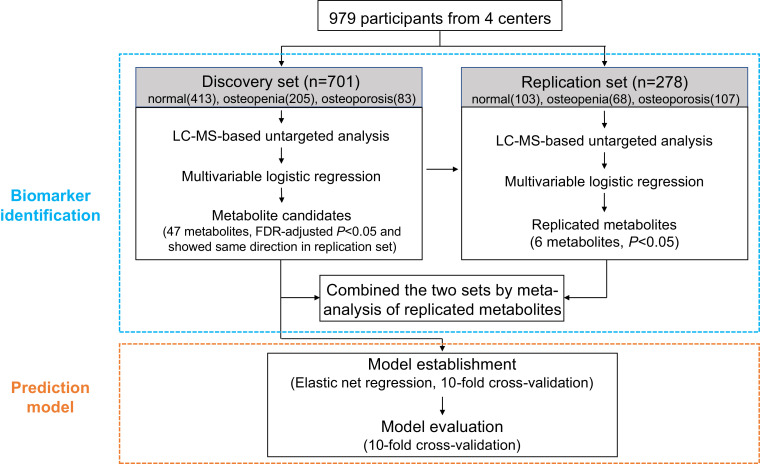
Study design. FDR, false discovery rate.

From March 2015 to November 2018, participants who visited our four clinical centres in southeast China were invited to participate in our study. The exclusion criteria included: 1) participants younger than 18 years; 2) those had diseases affecting bone metabolism or calcium absorption, such as fractures, endocrine system disease including diabetes and thyroid disorders, haematological diseases including leukaemia and myeloma, systemic lupus erythematosus, or renal disease; 3) those who received medications or therapy that may affect BMD, such as glucocorticoids and immunosuppressive agents, within three months before the study. In total, 979 participants who had spine or hip BMD measurements by DXA scans and donated blood samples within two days of DXA scans were included. In our analysis, these participants were divided according to their recruitment dates into a discovery set (*n*=701, those recruited during March 2015 and October 2015 from all the four centres) and a replication set (*n*=278, those recruited during June 2018 and November 2018 and all of them were from Hangzhou centre only) (Fig. 1).

The demographic data were collected by trained nurses. Bodyweight and height were measured with light clothes. Body mass index (BMI) was calculated as weight (in kilograms) divided by height (in meters) squared. The BMD at the lumbar spine (L1 to L4) or hip regions were measured by DXA scans (GE Lunar, Madison, WI, USA) at each centre. All BMD measurements were performed by trained technicians using a standardized protocol. Daily quality-control scans were performed using the spine phantom. The T-scores for BMD at the spine and hip were calculated by comparing with the BMD of healthy young people of the same sex. Based on World Health Organization criteria on osteoporosis,[9] a T-score of -1 and above is defined as normal BMD, a T-score of -2.5 to -1 indicates osteopenia, and a T-score of -2.5 and below indicates osteoporosis.

### 2.2 Ethics statement

All participants provided informed consent, and the study was approved by the ethics committee at each centre (the Taizhou Hospital Affiliated to Wenzhou Medical University, the Second Affiliated Hospital of Shantou University Medical College, the Sir Run Run Shaw Hospital, and the Shaoxing People's Hospital; Reference number, 2017053).

### 2.3 Metabolomic profiling

Overnight fasting blood samples were collected into a vacuum tube and kept in a portable Styrofoam box with ice packs (0–4 °C) and were centrifugated within two hours. Immediately after centrifugation, plasma samples were stored at −80 °C. Untargeted metabolite detection and quantification were conducted by an LC-MS-based metabolomics platform in both the discovery and replication sets separately. All plasma samples, including the quality control samples, were mixed with 200 μl prepared internal standard methanol solution containing 4 μg/ml L-Phenylalanine (D8) (Cambridge Isotope Laboratories) by vortexing for 1 min. The coefficients of variation of the internal standard in all quality control samples were less than 0.2. Then the mixtures were under 4 °C for 5 min and centrifuged at 13,000 rpm for 15 min at 4 °C to precipitate the protein and extract the metabolites.

Plasma samples and QC samples were analyzed on Agilent 1290 Infinity IIUHPLC system and Agilent 6545 UHD and Accurate-Mass Q-TOF mass spectrometer with a Waters XBridge BEH Amide Column (2.5 μm, 100 × 2.1 mm). A 13 min gradient using mobile phase A (0.1% Formic acid and 10mM Ammonium formate in water) and mobile phase B (0.1% Formic acid in Acetonitrile) were applied. HPLC grade of acetonitrile and formic acid were obtained from Merck, and the ammonium formate was obtained from Sigma-Aldrich. An optimized elution gradient was as follows: 0–1 min, 95%B; 1–3 min, 95%-85%B; 3–13 min, 85%-60%B. The flow rate was set at 0.4 ml/min; the column temperature was set at 25 °C; the injection volume was 2 μl. The system was immediately returned to initial conditions within 5 min for column re-equilibration after a run. The mass data were acquired both in ESI+ and ESI- mode using a mass range from 50 to 1100. In ESI+ mode, the Capillary voltage was 4 KV, with Nozzle Voltage of 250 V, Gas Temp of 325 °C, Fragmentor Voltage of 120 V. In ESI- mode, the Capillary voltage was 3.5 KV, with Nozzle Voltage of 1500 V, Gas Temp of 325 °C, Fragmentor Voltage of 120 V. Reference ions, used to monitor the accuracy of the mass axis, were 121.0507, 922.0098 in ESI+ mode and 112.9856, 1033.9881in ESI- mode. In the sequence, QC samples and blank samples were analyzed at intervals of 10 study samples.

The Agilent Masshunter Qualitative Analysis B.07.00 was used to convert the raw mass data to the mzData format. The extraction, alignment, and integration of the peaks were performed using the open-source R software package XCMS. The nearest QC normalization was performed after the IS normalization. The IS normalized values were calculated using the ratio of all the features’ values in plasma samples and QC samples over the IS quasi-molecular ion ([M+H] + in ESI+ mode and [M-H] - in ESI- mode) multiplied by the mean values of IS ion in all plasma samples and QC samples. Then the standardized values were calculated using the ratio of the IS normalized values in each plasma sample over the value of the same feature in the nearest QC sample multiplied by the median value measured across the QC samples.

We identified the features based on matching the accurate m/z value obtained from the metabolomics analysis and the MS/MS fragments obtained from the QC sample with those of entries in the Metlin and HMDB database. After combining the ESI+ and ESI- modes, some metabolites will have multiple adductions. The value of ion with the lowest variance for the QC samples was preferred as the value of the metabolite. Eventually, 403 metabolites were detected with known identification, including 61 in amino acids pathway, 17 carbohydrates, 4 cofactors and vitamins, 28 in energy metabolism pathway, 231 lipids, 12 nucleotides, 21 peptides, and 29 others. Plasma CTX-I levels were measured using an ELISA kit (Serum CrossLaps® (CTX-I), IDS, Shanghai, China).

### 2.4 Statistical analysis

In the analysis of clinical characteristics, participants were divided into three groups, i.e., the reference group (both the spine and hip T-scores were over -1), osteoporosis group (the T-score for spine or hip was below -2.5), and the others as osteopenia group. Clinical characteristics across groups were compared using analysis of variance for continuous variables and chi-square tests for categorical variables.

Metabolites with >20% of missing values (*n*=118) or a large coefficient of variation (>2.5, *n*=20) were removed before analysis, and for the remaining metabolites, their missing values were imputed with the half detectable minimum value of the metabolite in the study populations. A rank-based inverse normal transformation was applied to the metabolomics data in order to approximate the normal distribution of metabolite levels for the remaining 265 metabolites in the discovery and replication set, respectively.[10] In clinical practice, the BMD status may have more important and straightforward implications than a quantitative trait. For the primary analyses, we combined participants from the osteopenia and the osteoporosis groups as the low BMD group and compared the metabolite levels in the low BMD group to that in the reference group. Multivariable logistic regression models with adjustment of age, sex, BMI, and centres were used to examine the relationship of each metabolite with BMD status in the discovery and replication sets, separately. The Benjamini-Hochberg false discovery rate (FDR) method was used for multiple testing adjustment in the discovery set accounting for 265 named metabolites. For the successfully replicated metabolites, their levels were further analyzed as quartiles (using cut-points defined in the reference group). The odds ratio (OR) and 95% confidence intervals (CIs) were calculated separately in each set and then were pooled by using an inverse variance-weighted meta-analysis with the fixed-effects model. To test the linear trend across quartiles, metabolites were analyzed as a continuous variable. In order to assess the composite association of these metabolites, a protective metabolite score of low BMD was calculated as the weighted sum of the levels of these metabolites. The weight for each metabolite was the regression coefficient for a 1-SD increment in the plasma levels of metabolite estimated from the multivariable logistic regression model. Multivariable linear regression models with adjustment of the above-mentioned factors were used to examine the relationship of selected metabolites with the quantitative spine BMD in the discovery and replication sets. A sensitivity analysis was conducted by further adjusting for menopausal status, a risk factor of osteoporosis in elderly women, to evaluate whether it affected the association of the metabolites with low BMD.

Multivariate data analysis was performed by using SIMCA-P+ (v12.0, Umetrics, Sweden). Partial least squared discriminant analysis (PLS-DA), a commonly-used multivariate analysis method in metabolomics studies,[11,12] was conducted with 7-fold cross-validation (CV) and Pareto variance scaling to assess the ability of the global metabolites in classifying the BMD status. The statistical significance of the PLS-DA model was tested with CV-ANOVA (*P* < 0.05).[13]

We assessed the ability of the global metabolomic profile for classification of low BMD group from the reference group using logistic regression with elastic net penalty implemented in the R package “glmnet” (*α* = 0.5)[14]. This analysis was limited in the discovery set, because the metabolites from discovery set and replication set were profiled in separate batches and there is a large batch effect of untargeted metabolome profiling.[15] As a combination of the Lasso and Ridge penalties, the elastic regression model is a regularized regression to avoid overfitting and improve predictive performance. 10-fold cross-validation (nfolds=10 in cv.glmnet function of the “glmnet”) was performed to identify the optimal value of the tuning parameter (λ), which yielded the minimum mean-squared error (minMSE).[14] The prediction accuracy based on lambda.1se (more conservative) and lambda.min (optimal output from the model) parameters were both assessed. Because the results using these two parameters were similar, only the results for lambda.min were presented. The predictive model scores were calculated as the weighted sum of all covariates with weights equal to the regression coefficients from the predictive models. The OR of low BMD per 1-SD increase of predictive model score were calculated by logistic regression models. The OR attributed to metabolites selected by the model was calculated by taking the metabolite score as the weighted sum of selected metabolite, with weights equal to the coefficients from the model, and then estimate the OR due to the metabolite score. 10-fold cross-validation was used to obtain an unbiased estimate of prediction accuracy. The area under the receiver operator characteristic (ROC) curves (AUCs) was computed using the predicted probability and the true status of low BMD for each sample. The DeLong test was used to compare the AUCs of different models.[16]

Because sex hormones exert an important influence on bone metabolism,[17] a stratified analysis by sex was further performed as a secondary analysis. A two-sided *P* < 0.05 was considered as statistical significance unless otherwise indicated. All the statistical analyses except for the PLS-DA were performed using R version 3.5.1 (https://www.r-project.org/).

### 2.5 Role of funding source

None of the funders has played any role in data collection, analysis, interpretation, or writing the report.

## 3 Results

### 3.1 Characteristics of the study population

Table 1 shows the characteristics of the study population across groups of normal BMD, osteopenia, and osteoporosis in the discovery and replication sets. In both sets, the participants in the osteoporosis and osteopenia groups were more likely to be older, women, menopausal, and lower BMI and higher spine BMD. The plasma CTX-I levels were higher in the osteopenia and osteoporosis groups compared to those in the normal BMD group in the discovery set.

**Table 1:** Baseline characteristics of study participants

	Discovery set (n=701)			Replication set (n=278)		
	Normal BMD (n=413)	Osteopenia (n=205)	Osteoporosis (n=83)	Normal BMD (n=103)	Osteopenia (n=68)	Osteoporosis (n=107)
Age, years	52.9 ± 12.0	59.0 ± 10.8	63.0 ± 9.1	62.6 ± 12.7	66.5 ± 13.9	70.3 ± 9.5
Women, n (%)	193 (46.7)	117 (57.1)	59 (71.1)	45 (43.7)	53 (77.9)	92 (86.0)
Menopause, n (%)	88 (45.6)	103 (88.0)	59 (100.0)	32 (71.1)	49 (92.5)	90 (97.8)
BMI, kg/m^2^	24.7 ± 3.2	24.2 ± 3.3	22.8 ± 2.9	24.3 ± 3.7	23.2 ± 3.2	22.4 ± 3.7
CTX-I, ng/ml	0.3 ± 0.2	0.3 ± 0.3	0.4 ± 0.3	-	-	-
Spine BMD, g/cm^2^	1.2 ± 0.1	0.9 ± 0.1	0.8 ± 0.1	1.2 ± 0.2	0.9 ± 0.1	0.7 ± 0.1
Hip BMD, g/cm^2^	1.1 ± 0.1	1.1 ± 0.2	1.1 ± 0.1	-	-	-

Values are means ± SDs unless otherwise indicated. Menopause information missing in 24 participants in the discovery set and 1 in the replication set. CTX-I missing in 46 participants in the discovery set. Spine BMD missing in 72 participants in the discovery set. Hip BMD missing in 606 participants in the discovery set.

*P* values were from analysis of variance for continuous variables and chi-square test for categorical variables. All *P* values except for hip BMD were <0.05.

BMI, body mass index; CTX-I, C-terminal telopeptide of type I collagen; BMD, bone mineral density.

### 3.2 Metabolites associated with low BMD

**Fig. 2:**
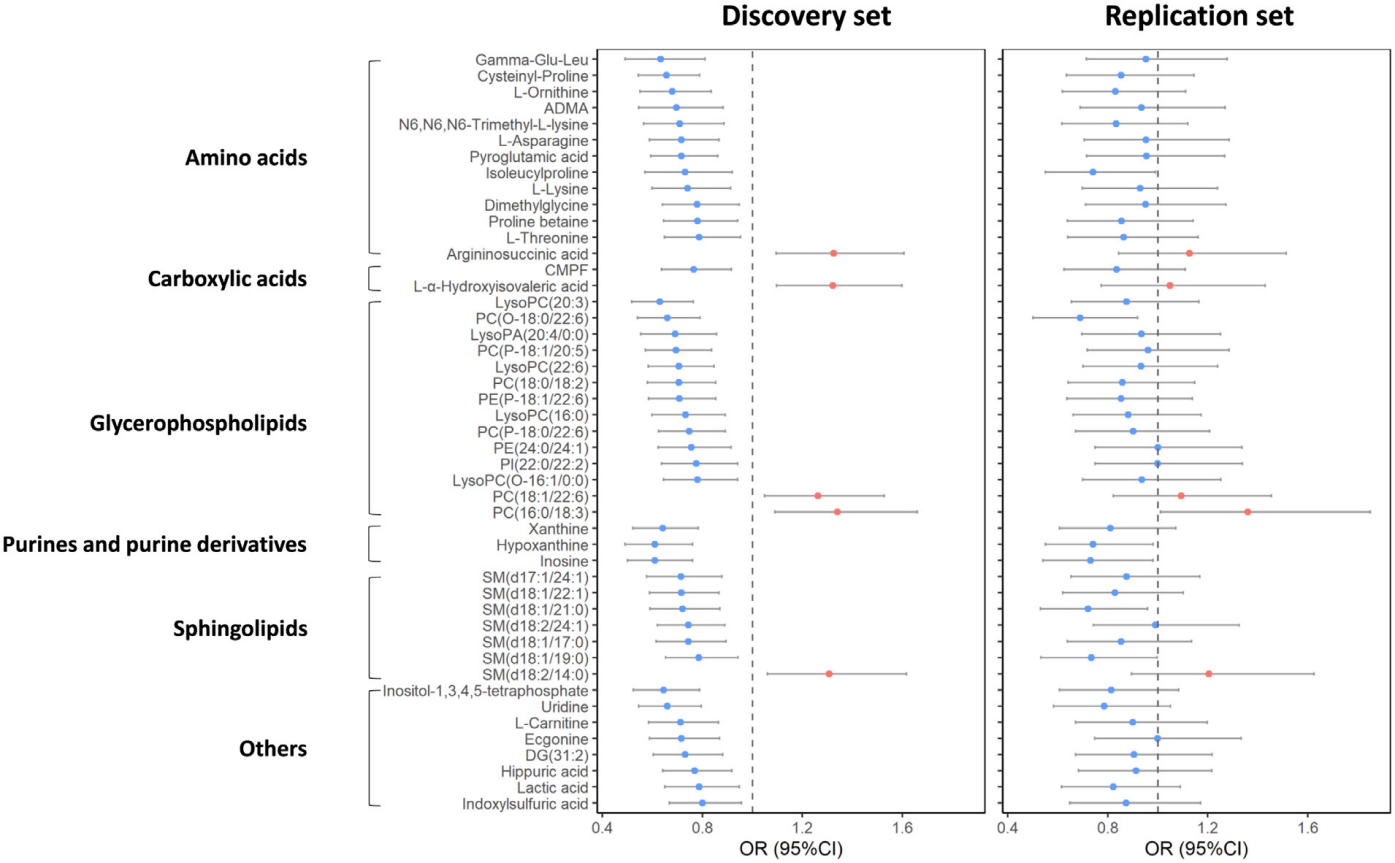
Metabolites demonstrating significant association with low BMD in the discovery set and maintaining the same change trend in the replication set. ORs (95% CIs) per 1-SD were obtained from the logistic regressions after adjusted for age, sex, and body mass index, further adjusted for centres in the discovery set. Metabolites in red indicate positive associations with low BMD, while in blue indicate inverse associations. CMPF, 3-Carboxy-4-methyl-5-propyl-2-furanpropionic acid; BMD, bone mineral density; OR, odds ratio; and CI, confidence interval.

**Fig. 3:**
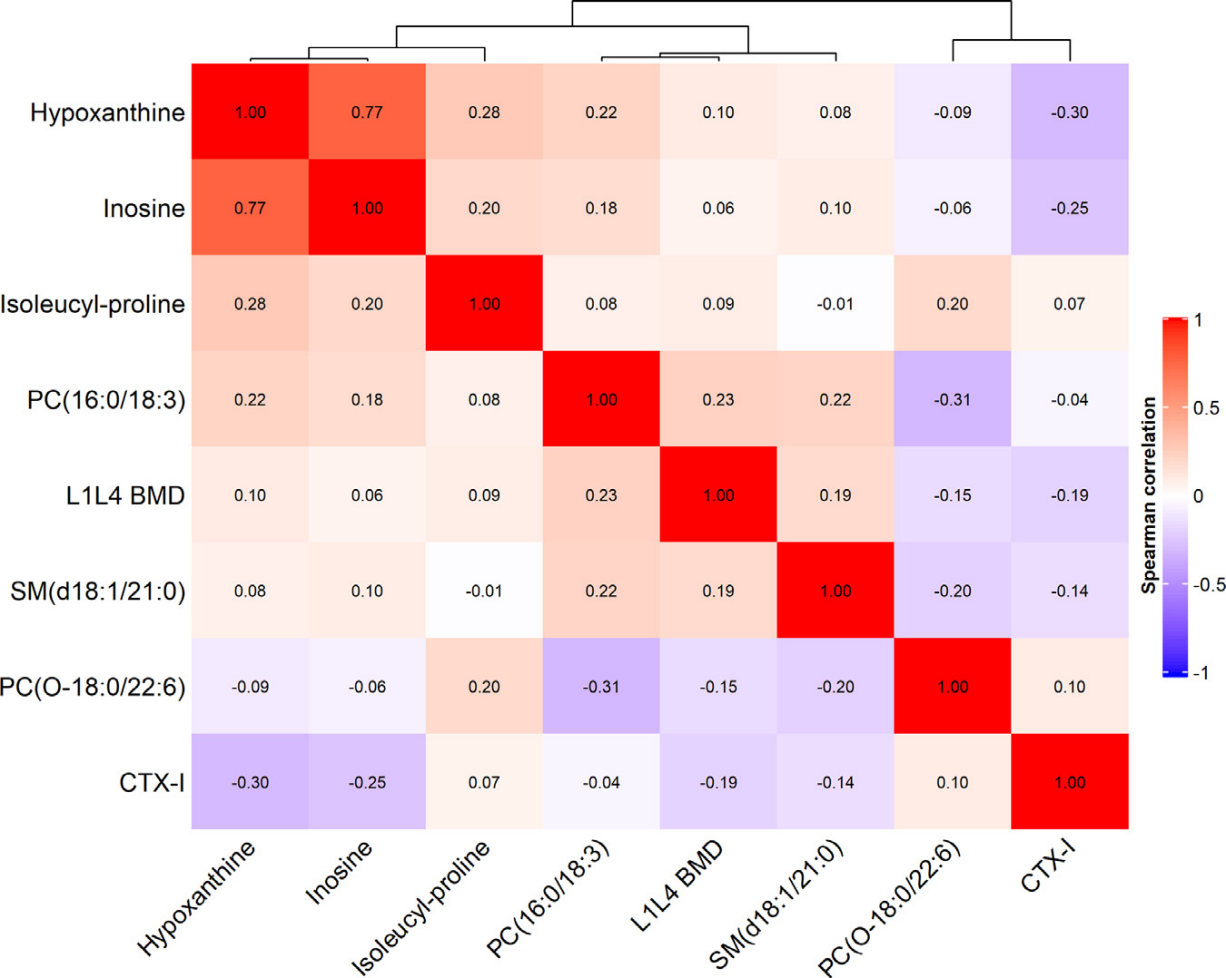
Spearman correlation heatmap of six selected metabolites, L1L4 BMD, and CTX-I in the discovery set. CTX-I, C-terminal telopeptide of type I collagen.

Through the analysis of individual metabolites as the continuous variables, 47 metabolites (13 amino acids, 2 carboxylic acids, 14 glycerophospholipids, 3 purines and purine derivatives, 7 sphingolipids, and 8 others) were significantly associated with low BMD status (FDR-adjusted *P* < 0.05 [multivariable logistic regression model]) in the discovery set and associated with low BMD in the same direction in the replication set (Fig. 2 and **Supplementary Table 1**). Among these metabolites, the levels of 5 metabolites were associated with a higher prevalence of low BMD status (OR per 1-SD ranged from 1.26 to 1.34), while most metabolites were associated with a decreased prevalence of low BMD status (ORs per 1-SD ranged from 0.61 to 0.80). In addition, six of these metabolites (i.e., inosine, hypoxanthine, PC (O-18:0/22:6), SM (d18:1/21:0), isoleucyl-proline, and PC (16:0/18:3)) retained their significant associations with low BMD in the replication set (Table 2). Among these six metabolites, the Spearman correlation coefficients of their levels ranged from -0.26 (between PC (O-18:0/22:6) and PC (16:0/18:3)) to 0.81 (between hypoxanthine and inosine), and none was in a close correlation with CTX-I (Fig. 3). PC (16:0/18:3) was associated with a higher prevalence for low BMD (OR [95% confidence interval (CI)]: 1.34 [1.09–1.66] in the discovery set and 1.36 [1.01–1.85] in the replication set). The other five metabolites were associated with a lower prevalence for low BMD (OR ranging from 0.61 to 0.74 per SD). Participants in the top quartile of plasma PC (16:0/18:3) levels had a more than doubled odds of low BMD compared to those in the bottom quartile in both the discovery (OR: 2.57, 95% CI: 1.42–4.70) and replication (OR: 2.30, 95% CI: 0.98–5.47) sets. While for the rest five metabolites, the participants in the top quartile of individual plasma metabolites had 47% to 67% reduced odds of low BMD, and the largest effect size was seen for PC (O-18:0/22:6). In the meta-analysis combining the discovery and replication sets together, the adjusted OR (95%CIs) of low BMD associated with a 1-SD increase of the metabolites were for inosine 0.65 (0.55–0.77), for hypoxanthine 0.65 (0.55–0.78), for PC (O-18:0/22:6) 0.66 (0.57–0.78), for SM (d18:1/21:0) 0.72 (0.61–0.84), for isoleucyl-proline 0.73 (0.61–0.88), and for PC (16:0/18:3) 1.35 (1.13–1.60) (Table 2).

**Table 2:** The associations between selected metabolites and low BMD in discovery and replication sets

	Inosine	Hypoxanthine	PC (O-18:0/22:6)	SM (d18:1/21:0)	Isoleucyl-proline	PC (16:0/18:3)	Metabolite score
**Discovery set (n=701)**							
Per 1-SD Increment	0.61 (0.50-0.76)	0.61 (0.49-0.76)	0.66 (0.54-0.79)	0.72 (0.59-0.87)	0.73 (0.57-0.92)	1.34 (1.09-1.66)	0.57 (0.49-0.67)
First quartile	1.00 (referent)	1.00 (referent)	1.00 (referent)	1.00 (referent)	1.00 (referent)	1.00 (referent)	1.00 (referent)
Second quartile	1.13 (0.71-1.81)	0.67 (0.42-1.06)	0.70 (0.44-1.11)	0.50 (0.30-0.82)	1.47 (0.91-2.37)	1.38 (0.86-2.24)	0.36 (0.22-0.59)
Third quartile	0.72 (0.44-1.18)	0.57 (0.34-0.92)	0.33 (0.19-0.54)	0.53 (0.32-0.87)	0.75 (0.45-1.27)	1.24 (0.72-2.14)	0.46 (0.28-0.76)
Fourth quartile	0.39 (0.22-0.70)	0.36 (0.20-0.64)	0.37 (0.22-0.62)	0.42 (0.25-0.70)	0.46 (0.23-0.90)	2.57 (1.42-4.70)	0.16 (0.09-0.30)
*P* for trend	5.98 × 10^−6^	5.96 × 10^−6^	9.25 × 10^−6^	7.95 × 10^−4^	0.007	0.007	2.89 × 10^−11^
**Replication set (n=278)**							
Per 1-SD Increment	0.73 (0.54-0.98)	0.74 (0.55-0.98)	0.69 (0.50-0.92)	0.72 (0.53-0.96)	0.74 (0.55-0.99)	1.36 (1.01-1.85)	0.63 (0.49-0.80)
First quartile	1.00 (referent)	1.00 (referent)	1.00 (referent)	1.00 (referent)	1.00 (referent)	1.00 (referent)	1.00 (referent)
Second quartile	0.54 (0.23-1.24)	0.57 (0.25-1.31)	0.46 (0.21-1.00)	0.46 (0.20-1.03)	1.08 (0.52-2.25)	1.92 (0.83-4.49)	0.99 (0.45-2.20)
Third quartile	1.03 (0.47-2.24)	1.11 (0.51-2.42)	0.39 (0.17-0.87)	0.68 (0.30-1.53)	0.50 (0.21-1.17)	1.86 (0.78-4.48)	0.62 (0.28-1.36)
Fourth quartile	0.38 (0.17-0.85)	0.38 (0.17-0.84)	0.33 (0.14-0.77)	0.53 (0.24-1.14)	0.49 (0.21-1.12)	2.30 (0.98-5.47)	0.30 (0.13-0.69)
*P* for trend	0.04	0.04	0.02	0.03	0.05	0.04	1.71 × 10^−4^
**Combined set (meta)**							
Per 1-SD Increment	0.65 (0.55-0.77)	0.65 (0.55-0.78)	0.66 (0.57-0.78)	0.72 (0.61-0.84)	0.73 (0.61-0.88)	1.35 (1.13-1.60)	0.59 (0.52-0.68)
First quartile	1.00 (referent)	1.00 (referent)	1.00 (referent)	1.00 (referent)	1.00 (referent)	1.00 (referent)	1.00 (referent)
Second quartile	0.95 (0.63-1.43)	0.65 (0.43-0.97)	0.63 (0.42-0.94)	0.49 (0.32-0.74)	1.34 (0.90-2.00)	1.50 (0.99-2.28)	0.48 (0.31-0.73)
Third quartile	0.80 (0.52-1.21)	0.69 (0.45-1.04)	0.34 (0.22-0.53)	0.57 (0.37-0.87)	0.67 (0.43-1.05)	1.39 (0.88-2.20)	0.51 (0.33-0.77)
Fourth quartile	0.39 (0.24-0.62)	0.37 (0.23-0.59)	0.36 (0.23-0.56)	0.45 (0.29-0.69)	0.47 (0.28-0.80)	2.48 (1.52-4.04)	0.20 (0.12-0.33)
*P* for trend	1.00 × 10^−6^	1.07 × 10^−6^	4.44 × 10^−7^	6.14 × 10^−5^	8.31 × 10^−4^	7.66 × 10^−4^	2.69 × 10^−14^

Inverse normal transformation was applied to raw values of metabolites. The metabolite score was calculated by a weighted sum of concentrations of 5 metabolites. Values are odds ratio (95% confidence intervals) for low BMD from logistic regressions. All models were adjusted for age, sex, and body mass index, further adjusted for centres in the discovery set. BMD, bone mineral density; SD, standard deviation.

We also examined the association of the 6 successfully replicated metabolites with the quantitative spine BMD among the subgroup of participants with available data. The fully-adjusted associations of quantitative spine BMD in the combined samples were consistent with our main results of BMD status, although in separate set a few metabolites failed to reach statistical significance which was partially due to a smaller sample size (**Supplementary Table 2**). A sensitivity analysis further adjusted for menopausal status showed that the associations between selected 6 metabolites and low BMD did not change materially (**Supplementary Table 3**).

The metabolite score calculated from the six selected metabolites was significantly associated with decreased odds of low BMD. In the meta-analysis, individuals in the top quartile of the metabolite score had 80% reduced odds of low BMD compared with those in the bottom quartile (OR, 0.20, 95%CI: 0.12–0.33; *P* for trend <0.001 [multivariable logistic regression model]), and the OR of low BMD associated with a 1-SD increment of the metabolite score was 0.59 (95%CI: 0.52–0.68) (Table 2). Among men, DG (31:2) was associated with decreased odds of low BMD, the ORs (95%CIs) were 0.68 (0.49–0.92) and 0.51 (0.28–0.87) in discovery and replication sets, respectively. While among women, SM (d18:1/24:1) was associated with decreased odds of low BMD with ORs (95%CIs) 0.71 (0.53–0.95) and 0.66 (0.44–0.98) in discovery and replication sets, respectively.

### 3.3 Performance of metabolites for low BMD classification

**Fig. 4:**
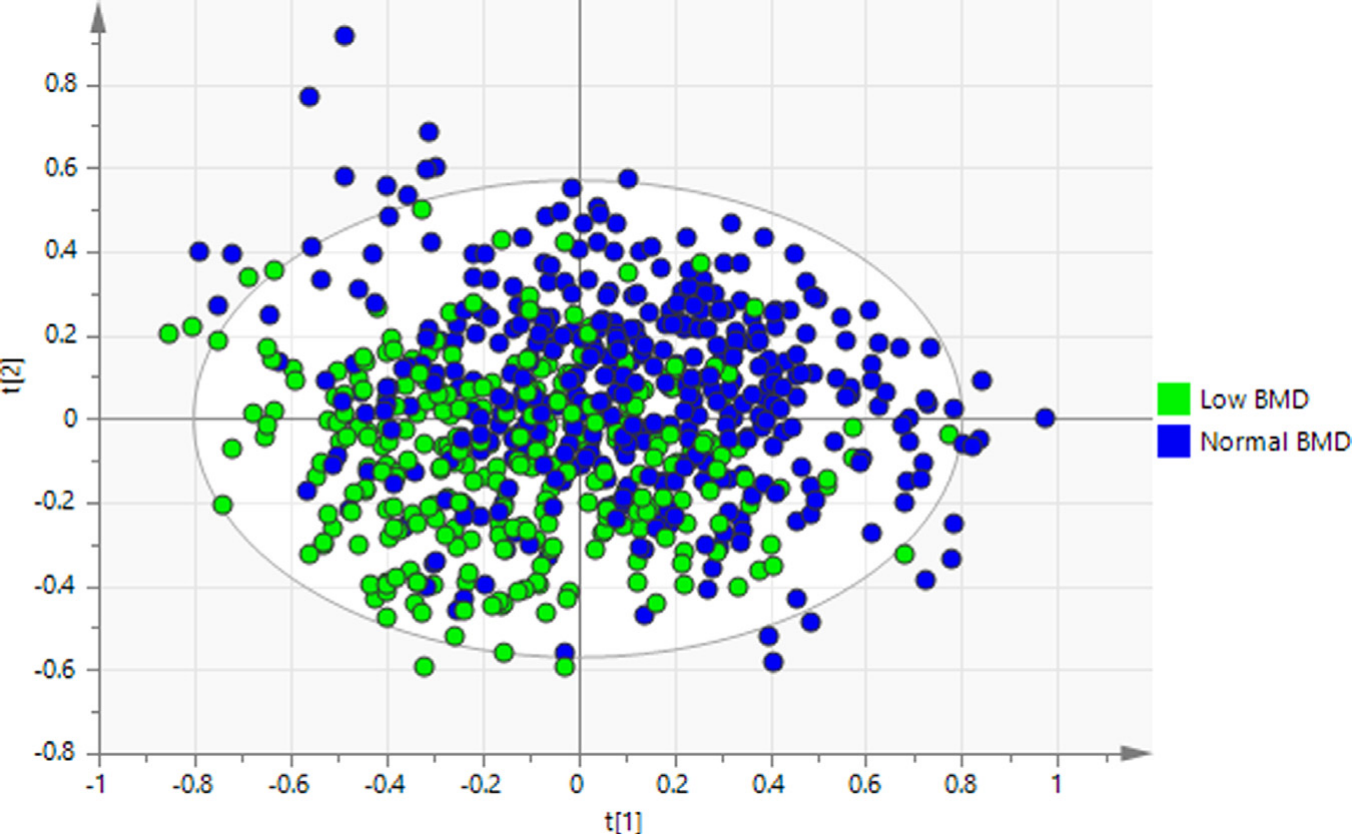
Partial least square discriminant analysis model to classify the low and normal BMD groups. *P* = 1.68 × 10^−32^ estimated from CV-ANOVA. BMD, bone mineral density.

**Fig. 5:**
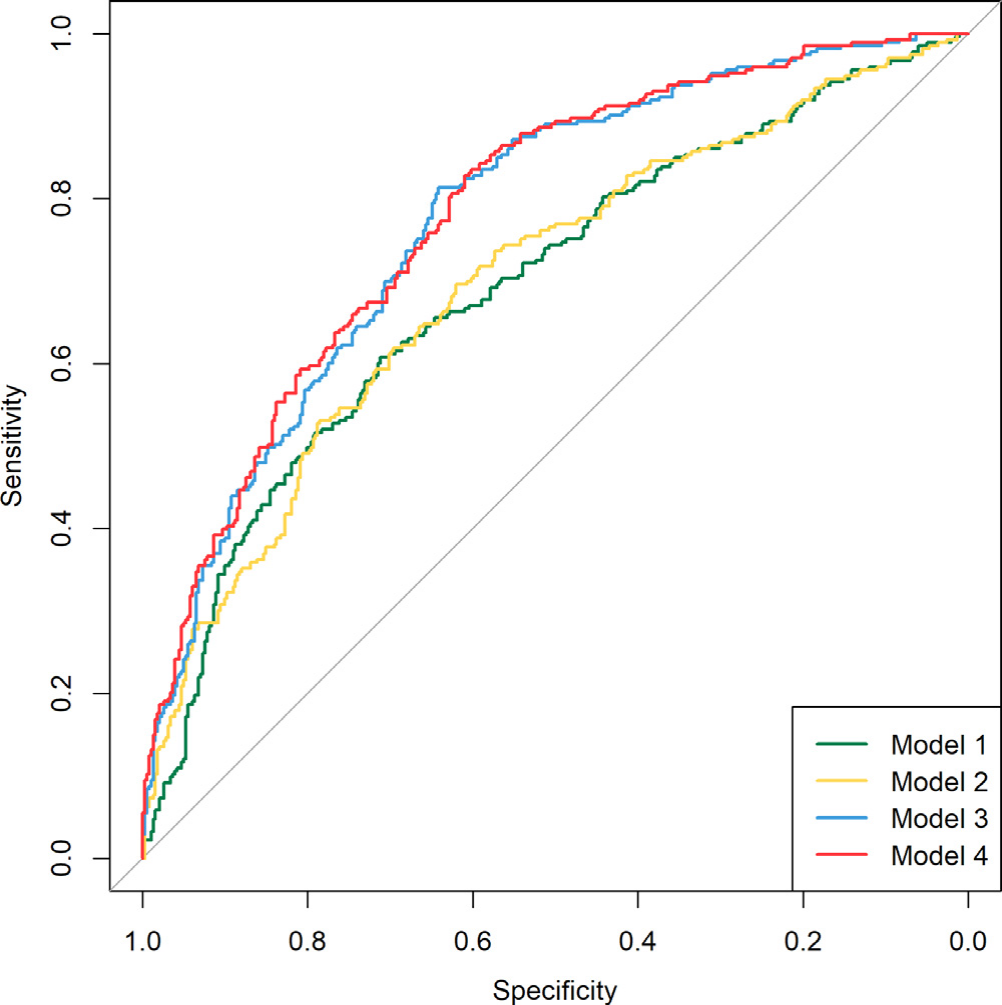
ROC curves for the distinguish of low BMD from normal BMD. Model 1 was the basic model including age, sex, and BMI, AUC = 0.693 (95% CI: 0.651–0.734); model 2 including age, sex, BMI, and CTX-I, AUC = 0.698 (95% CI: 0.657–0.739); model 3 including age, sex, BMI, and selected metabolites, AUC = 0.776 (95% CI: 0.741–0.812); and model 4 including age, sex, BMI, CTX-I, and selected metabolites, AUC = 0.782 (95% CI: 0.748–0.817). BMD, bone mineral density; BMI, body mass index; CTX-I, C-terminal telopeptide of type I collagen; ROC, receiver operating curve; AUC, area under the receiver operating curve; and CI, confidence interval.

The metabolic profiles of the low BMD were significantly different from those of normal BMD (Fig. 4). To assess the performance of the selected metabolites from the elastic model for low BMD status, the AUCs were calculated in 4 hierarchical models containing the traditional risk factors (age, sex, and BMI), one clinical biomarker, i.e., CTX-I, and those selected metabolites. The coefficients of full models after running the elastic net model 10 times were shown in **Supplementary Table 4**. Model 1, i.e., the basic model, included age, sex, and BMI, with an AUC1 of 0.693 (95% CI: 0.651–0.734). CTX-I did not improve the predictability (AUC2, 0.698; 95% CI: 0.657–0.739 in model 2, and *P*=0.43 comparing AUC2 with AUC1 [DeLong test]). Introduction of selected metabolites to both model 1 (model 3: AUC3, 0.776; 95% CI: 0.741–0.812; *P*=0.003 comparing AUC3 with AUC1 [DeLong test]) and to model 2 (model 4: AUC4, 0.782; 95% CI: 0.748–0.817; *P*=0.002 comparing AUC4 with AUC2 [DeLong test]) significantly improved the predictability. CTX-I alone did not improve the predictability in the model consisting of traditional risk factors and selected metabolites (*P*=0.18 comparing AUC4 with AUC3 [DeLong test]) (Fig. 5). We also calculated the ORs of low BMD per 1-SD increase of predictive model score. The ORs were 1.17 (95% CI: 1.01–1.38) for the traditional model (aforementioned model 2: traditional risk factors + CTX-I), and 5.94 (95% CI: 4.54–7.95) for the full model (aforementioned model 4: traditional risk factors + CTX-I + selected metabolites), with OR=4.45 (95% CI: 3.50–5.77) attributed to the selected metabolites (Table 3).

**Table 3:** Selected models and odds of low BMD

	Variables in model	OR (95% CI) per 1-SD increase of predictive model score	*P*
Traditional model	Age, sex, BMI, centres, and CTX-I	1.17 (1.01-1.38)	0.04
Full model	Age, sex, BMI, centres, CTX-I, and selected metabolites	5.94 (4.54-7.95), among which 4.45 (3.50-5.77) was attributed to the selected metabolites	<0.001

The predictive model scores were calculated as the weighted sum of all covariates with weights equal to the regression coefficients from the predictive models built by the logistic regression model or the elastic net regression models.

BMD, bone mineral density; BMI, body mass index; CTX-I, C-terminal telopeptide of type I collagen; OR, odds ratio; CI, confidence interval; and SD, standard deviation.

## 4 Discussion

Using a mass spectrometry-based metabolite profiling platform, we identified and validated a panel of six metabolites (i.e., inosine, hypoxanthine, PC (O-18:0/22:6), SM (d18:1/21:0), isoleucyl-proline, and PC (16:0/18:3)) associated with the BMD status in two independent Chinese populations. The composite score of these six metabolites was associated with 80% lower odds of low BMD status across populations with extreme score quartiles. Furthermore, selected metabolites significantly improved the prediction performance for low BMD status beyond traditional risk factors and clinical biomarkers such as CTX-I.

In our study, we identified and validated the associations of 6 potential metabolite biomarkers from the globally profiled circulating metabolites with BMD status, in a total of 979 Chinese participants. Though a few recent studies have used metabolomics profiling as a discovery tool for osteoporosis biomarkers, most used a relatively less-resolution technique or in a smaller population.[18], [19], [20], [21], [22], [23] You and colleagues measured plasma metabolome using proton nuclear magnetic resonance spectroscopy (^1^H NMR) among 601 healthy Taiwanese women, and found circulating metabolome data could differentiate the BMD status.[21] Miyamoto and colleagues detected 57 serum metabolites utilizing capillary electrophoresis-MS among 57 postmenopausal women, and found that the levels of hydroxyproline, Gly-Gly, and cystine were associated with BMD status.[19] Most recently, the TwinsUK study found 15 metabolites were associated with BMD in a large population primarily comprised of women, and replicated four causally associated metabolites in a Chinese Hong Kong population.[23] Of note, the differences in metabolomics platforms (LC-MS vs. NMR vs. capillary electrophoresis-MS), statistical analyses, and differences in lifestyle factors and genetic architecture could also account for the discrepancy between our study and the others, at least to a certain degree.

To the best of our knowledge, the current study is the first showing that circulating global metabolomic profiling could effectively improve the classification performance of BMD status beyond a clinical risk factor as well as an established biomarker. The panel of selected metabolites from elastic net regression model, which provided good performance in both predictive accuracy and sparsity for the high-dimensional datasets,[24] significantly improved the classification performance of BMD status in our population, while the clinical bone turnover biomarker, i.e., CTX-I, failed the mission. In addition, the odds ratio of low BMD yield from the predictive model was largely attributed to the selected metabolites. Our findings are in line with those from a recent study, which indicated selected metabolites improved the performance of a classification model using bone turnover markers for osteoporosis,[20] though the model missed important demographic factors for osteoporosis such as age and weight.[25] Our findings provided the potential that a simple blood test of circulating metabolites may provide biomarker panels for mass screening in a clinic for easy detection of low BMD, independent of radiology resources.

Among the potential metabolite biomarkers from our study, both hypoxanthine and inosine are metabolites in the purine metabolic pathway and precursors of uric acid.[26] Their inverse associations with low BMD status in our populations are in line with the association of higher serum uric acid levels with lower risks of osteoporosis and fractures in previous studies.[27], [28], [29], [30], [31] In our discovery set, plasma uric acid levels were inversely associated with low BMD status (OR per SD: 0.81, 95% CI: 0.66–0.98), although not significant in the replication population (OR per SD: 0.92, 95% CI: 0.68–1.24). Uric acids are antioxidants capable of scavenging superoxide, and they block the formation of peroxynitrite,[32] which may help to prevent osteoporosis. However, whether the associations of uric acids with BMD are causal or independent of body fat is unclear yet.[27,[33], [34], [35]] To our best knowledge, our study is the first to report the associations of uric acid precursors, hypoxanthine, and inosine with BMD status,[18], [19], [20], [21], [22], [23] highlighting the importance of the purine metabolic pathway in bone metabolism.

Both PC (16:0/18:3) and PC (O-18:0/22:6) are phosphatidylcholines, and they were associated with BMD in opposite directions. Similar to our results, a previous study also found phosphatidylcholines were associated with BMD in various directions among Chinese postmenopausal women.[20] PC (16:0/18:3) consists of one chain of palmitic acid which induced osteoclastogenesis[36] and impaired osteoblast activity,[37] and another chain of γ-linolenic acid, which is related to higher odds of low BMD status.[22] In our study, palmitic acid levels were associated with increased odds of low BMD in women, but not in men. Recent studies suggested linoleic acid was correlated with lower BMD levels[22,38] and associated with increased odds of hip fracture.[38] PC (O-18:0/22:6) consists of one chain of stearyl alcohol and one chain of an omega-3 polyunsaturated fatty acid, i.e., docosahexaenoic acid (DHA), which inhibited osteoclastogenesis from *in vitro* studies.[39,40] Observational evidence suggested that both higher consumption levels and blood levels of DHA were associated with BMD levels in women and men,[41,42] however interventional studies did not provide positive evidence.[43]

SM (d18:1/21:0) is a type of sphingolipids which are composing biological membranes. Sphingolipids play a role in signal transduction and may regulate bone remodelling.[44] Our finding of SM (d18:1/21:0) was consistent with a recent smaller study, demonstrating that two sphingolipids were associated with BMD status in men only.[20] Of note, in our sex-specific analysis, another sphingolipid SM (d18:1/24:1) was suggestively associated with BMD status in our female participants only (*P*=0.03 and 0.04 in discovery and replication set, respectively). Circulating isoleucyl-proline may exert antioxidant effects,[45] and it may explain its association with BMD in our study to some extent. Lactic acid, which was associated with BMD in our discovery set only, has been previously reported to be associated with a decreased risk of low BMD in Taiwanese women.[21] Lactate may regulate the collagen biosynthesis in osteogenesis and thus increase bone formation.[21,46] Besides, threonine levels were inversely associated with low BMD in our discovery set, and this is in line with the findings from two recent metabolomic studies in U.S. Caucasian and Chinese.[18,20]

Gender disparities exist in osteoporosis and bone loss. Women tend to have a younger onset of bone loss and lose bone at a faster rate compared to men.[47] Deficiency of sex hormones, especially estrogen, plays an important role in the bone loss for both genders, and compared to men its influence is more pronounced for women at younger ages.[47] In the current study, the level of DG (31:2) was negatively associated with low BMD in men but not in women, and the level of SM (d18:1/24:1) was associated with low BMD in women but not in men. DG (31:2), a member of the family of diacylglycerols, and its beneficial effect on bone mass might be from the increased differentiation of bone marrow cells into an osteogenic instead of adipogenic lineage.[48]

Several limitations in our study warrant mention. First, we did not collect the dietary habits, nutritional supplements, and other lifestyle information of the participants, which would influence the circulating metabolites as well as the BMD level. Second, with a cross-sectional design, our study could not infer the causal relationship between the plasma metabolites and BMD. Third, as the untargeted metabolomic profiling was conducted in the discovery and replication sets separately without duplicate reference samples, it was challenging to combine the two sets or validate the prediction model from the discovery set in the replication set.

In conclusion, our findings demonstrated that six plasma metabolites were associated with BMD status, providing clues to relevant mechanisms in osteoporosis. In addition, a panel of plasma metabolites provided the potential for early diagnosis of low BMD beyond the clinical index in the current practice. Our study highlights the value of metabolomic profiling in the biomarker discovery for osteoporosis prevention and diagnosis.

## Contributors

X.D., A.Q., L.L., and Y.Z. designed the study. Y.Q., D.H., Z.X., G.Y., and A.Q. collected the clinical data and plasma samples. Z.M., X.D., S.G., J.H., and L.L. conducted data analyses. Z.M., X.D., Y.Q. and Y.Z. interpreted the data and drafted the manuscript. Y.Z., L.L., and J.S. take responsibility for the integrity of the data analysis. All authors read and approved the final version of the manuscript, and have had access to the raw data.

## Declaration of Competing Interests

The authors have nothing to disclose. The authors declare no conflict of interest.

## Appendix Supplementary materials

Image, application 1
