# Supplementary material for: Association between the metabolome and bone mineral density in a Chinese population
Source: eBioMedicine. 2020 Nov 10;62:103111. doi: 10.1016/j.ebiom.2020.103111 (PMC7670189; doi:10.1016/j.ebiom.2020.103111)
Supplement: Supplementary file 1 [file mmc1.docx]

**Supplementary Table 1. Metabolites associated with low BMD in the discovery set and replication set**

| **Metabolites** | **Discovery set** | | | **Replication set** | |
| --- | --- | --- | --- | --- | --- |
|  | **OR (95% CI)** | ***P*** | **FDR-adjusted *P*** | **OR (95% CI)** | ***P*** |
| Hypoxanthine | 0.61 (0.49-0.76) | 5.96×10^-06^ | 3.96×10^-04^ | 0.74 (0.55-0.98) | 0.038 |
| Inosine | 0.61 (0.50-0.76) | 5.98×10^-06^ | 3.96×10^-04^ | 0.73 (0.54-0.98) | 0.037 |
| LysoPC(20:3) | 0.63 (0.52-0.76) | 3.57×10^-06^ | 3.96×10^-04^ | 0.87 (0.66-1.16) | 0.358 |
| Gamma-Glu-Leu | 0.63 (0.49-0.81) | 3.29×10^-04^ | 0.005 | 0.95 (0.71-1.28) | 0.747 |
| Xanthine | 0.64 (0.52-0.78) | 1.77×10^-05^ | 5.86×10^-04^ | 0.81 (0.61-1.07) | 0.144 |
| Inositol-1,3,4,5-tetraphosphate | 0.64 (0.52-0.79) | 2.48×10^-05^ | 7.29×10^-04^ | 0.81 (0.61-1.08) | 0.160 |
| Cysteinyl-Proline | 0.66 (0.54-0.79) | 8.58×10^-06^ | 4.09×10^-04^ | 0.85 (0.63-1.15) | 0.292 |
| PC(O-18:0/22:6) | 0.66 (0.54-0.79) | 9.25×10^-06^ | 4.09×10^-04^ | 0.69 (0.50-0.92) | 0.015 |
| Uridine | 0.66 (0.54-0.80) | 1.61×10^-05^ | 5.86×10^-04^ | 0.79 (0.58-1.05) | 0.106 |
| L-Ornithine | 0.68 (0.55-0.83) | 2.79×10^-04^ | 0.005 | 0.83 (0.62-1.11) | 0.215 |
| LysoPA(20:4/0:0) | 0.69 (0.55-0.86) | 8.58×10^-04^ | 0.008 | 0.93 (0.70-1.25) | 0.648 |
| PC(P-18:1/20:5) | 0.69 (0.57-0.84) | 1.60×10^-04^ | 0.004 | 0.96 (0.72-1.28) | 0.784 |
| ADMA | 0.70 (0.55-0.88) | 0.003 | 0.018 | 0.94 (0.69-1.27) | 0.666 |
| LysoPC(22:6) | 0.71 (0.58-0.85) | 2.30×10^-04^ | 0.005 | 0.93 (0.70-1.24) | 0.634 |
| PC(18:0/18:2) | 0.71 (0.58-0.85) | 3.74×10^-04^ | 0.006 | 0.86 (0.64-1.15) | 0.303 |
| PE(P-18:1/22:6) | 0.71 (0.58-0.85) | 3.21×10^-04^ | 0.005 | 0.85 (0.64-1.14) | 0.285 |
| N6,N6,N6-Trimethyl-L-lysine | 0.71 (0.56-0.89) | 0.003 | 0.017 | 0.83 (0.62-1.12) | 0.228 |
| L-Carnitine | 0.71 (0.58-0.86) | 6.63×10^-04^ | 0.007 | 0.90 (0.67-1.20) | 0.469 |
| SM(d17:1/24:1) | 0.71 (0.58-0.88) | 0.002 | 0.011 | 0.87 (0.65-1.17) | 0.366 |
| L-Asparagine | 0.72 (0.59-0.87) | 6.71×10^-04^ | 0.007 | 0.95 (0.71-1.28) | 0.750 |
| Pyroglutamic acid | 0.72 (0.59-0.86) | 4.57×10^-04^ | 0.006 | 0.95 (0.72-1.27) | 0.745 |
| Ecgonine | 0.72 (0.59-0.87) | 7.48×10^-04^ | 0.008 | 1.00 (0.75-1.33) | 0.997 |
| SM(d18:1/22:1) | 0.72 (0.59-0.87) | 7.19×10^-04^ | 0.008 | 0.83 (0.62-1.1) | 0.197 |
| SM(d18:1/21:0) | 0.72 (0.59-0.87) | 7.95×10^-04^ | 0.008 | 0.72 (0.53-0.96) | 0.028 |
| Isoleucylproline | 0.73 (0.57-0.92) | 0.007 | 0.032 | 0.74 (0.55-0.99) | 0.045 |
| DG(31:2) | 0.73 (0.60-0.88) | 0.001 | 0.010 | 0.90 (0.67-1.22) | 0.507 |
| LysoPC(16:0) | 0.73 (0.60-0.89) | 0.002 | 0.014 | 0.88 (0.66-1.17) | 0.388 |
| L-Lysine | 0.74 (0.60-0.91) | 0.005 | 0.025 | 0.93 (0.70-1.24) | 0.616 |
| SM(d18:2/24:1) | 0.74 (0.62-0.89) | 0.001 | 0.010 | 0.99 (0.74-1.32) | 0.950 |
| SM(d18:1/17:0) | 0.74 (0.61-0.90) | 0.002 | 0.013 | 0.85 (0.64-1.13) | 0.278 |
| PC(P-18:0/22:6) | 0.75 (0.62-0.89) | 0.001 | 0.010 | 0.90 (0.67-1.21) | 0.486 |
| PE(24:0/24:1) | 0.76 (0.62-0.91) | 0.004 | 0.022 | 1.00 (0.75-1.34) | 1.000 |
| CMPF | 0.77 (0.64-0.92) | 0.004 | 0.021 | 0.83 (0.63-1.11) | 0.215 |
| Hippuric acid | 0.77 (0.64-0.92) | 0.004 | 0.021 | 0.91 (0.68-1.22) | 0.535 |
| PI(22:0/22:2) | 0.78 (0.64-0.94) | 0.011 | 0.040 | 1.00 (0.75-1.34) | 0.998 |
| Dimethylglycine | 0.78 (0.64-0.95) | 0.012 | 0.045 | 0.95 (0.71-1.27) | 0.732 |
| LysoPC(O-16:1/0:0) | 0.78 (0.64-0.94) | 0.010 | 0.039 | 0.94 (0.70-1.25) | 0.652 |
| Proline betaine | 0.78 (0.64-0.94) | 0.010 | 0.039 | 0.85 (0.64-1.14) | 0.287 |
| SM(d18:1/19:0) | 0.79 (0.65-0.94) | 0.010 | 0.039 | 0.73 (0.53-1.00) | 0.052 |
| Lactic acid | 0.79 (0.65-0.95) | 0.012 | 0.045 | 0.82 (0.62-1.09) | 0.179 |
| L-Threonine | 0.79 (0.65-0.95) | 0.015 | 0.050 | 0.86 (0.64-1.16) | 0.334 |
| Indoxylsulfuric acid | 0.80 (0.67-0.96) | 0.015 | 0.050 | 0.87 (0.65-1.17) | 0.368 |
| PC(18:1/22:6) | 1.26 (1.05-1.53) | 0.015 | 0.050 | 1.09 (0.82-1.45) | 0.537 |
| SM(d18:2/14:0) | 1.31 (1.06-1.62) | 0.013 | 0.046 | 1.20 (0.90-1.62) | 0.222 |
| L-α-Hydroxyisovaleric acid | 1.32 (1.10-1.60) | 0.004 | 0.021 | 1.05 (0.77-1.43) | 0.757 |
| Argininosuccinic acid | 1.32 (1.09-1.61) | 0.004 | 0.021 | 1.13 (0.84-1.51) | 0.422 |
| PC(16:0/18:3) | 1.34 (1.09-1.66) | 0.007 | 0.031 | 1.36 (1.01-1.85) | 0.044 |

All models were adjusted for age, sex, and body mass index, and further adjusted for centers in the discovery set.

BMD, bone mineral density; OR, odds ratio; CI, confidence interval; FDR, false discovery rate; CMPF, 3-Carboxy-4-methyl-5-propyl-2-furanpropionic acid.

**Supplementary Table 2. The associations of selected metabolites with the quantitative spine BMD in discovery and replication sets**

| **Metabolites** | **Discovery set** | | **Replication set** | | **Combined set (meta)** | |
| --- | --- | --- | --- | --- | --- | --- |
|  | **Beta ± SE** | **P** | **Beta ± SE** | **P** | **Beta ± SE** | **P** |
| Inosine | 0.16 ± 0.04 | 5.69×10^-5^ | 0.11 ± 0.05 | 0.05 | 0.14 ± 0.03 | 9.04×10^-6^ |
| Hypoxanthine | 0.15 ± 0.04 | 2.07×10^-4^ | 0.09 ± 0.05 | 0.10 | 0.13 ± 0.03 | 7.42×10^-5^ |
| PC(O-18:0/22:6) | 0.15 ± 0.04 | 4.53×10^-5^ | 0.08 ± 0.05 | 0.16 | 0.12 ± 0.03 | 2.58×10^-5^ |
| SM(d18:1/21:0) | 0.13 ± 0.04 | 0.001 | 0.14 ± 0.05 | 0.01 | 0.13 ± 0.03 | 3.44×10^-5^ |
| Isoleucyl-proline | 0.04 ± 0.05 | 0.42 | 0.13 ± 0.05 | 0.02 | 0.08 ± 0.04 | 0.02 |
| PC(16:0/18:3) | -0.10 ± 0.04 | 0.02 | -0.09 ± 0.05 | 0.09 | -0.10 ± 0.03 | 0.004 |

Inverse normal transformation was applied to raw values of metabolites and spine BMD. Beta ± SE were estimated from multivariable linear regression after adjusted for age, sex, and body mass index, further adjusted for centers in the discovery set.

**Supplementary Table 3. Sensitivity analysis of the association between selected metabolites and low BMD in discovery and replication set.**

| **Metabolites** | **Discovery set** | | **Replication set** | |
| --- | --- | --- | --- | --- |
|  | **OR (95% CI)** | ***P*** | **OR (95% CI)** | ***P*** |
| Inosine | 0.68 (0.55-0.85) | 5.74×10^-4^ | 0.76 (0.56-1.01) | 0.07 |
| Hypoxanthine | 0.68 (0.54-0.84) | 4.41×10^-4^ | 0.76 (0.56-1.02) | 0.07 |
| PC(O-18:0/22:6) | 0.70 (0.57-0.85) | 2.61×10^-4^ | 0.71 (0.51-0.96) | 0.03 |
| SM(d18:1/21:0) | 0.71 (0.58-0.87) | 9.27×10^-4^ | 0.68 (0.50-0.92) | 0.01 |
| Isoleucyl-proline | 0.69 (0.54-0.88) | 0.003 | 0.74 (0.54-0.99) | 0.05 |
| PC(16:0/18:3) | 1.36 (1.10-1.70) | 0.005 | 1.36 (1.01-1.86) | 0.04 |

Values are OR (95% CI) for low BMD from logistic regressions. All models were adjusted for age, sex, body mass index, and menopausal status, further adjusted for centers in the discovery set.

BMD, bone mineral density; OR, odds ratio; CI, confidence interval

**Supplementary Table 4. Mean and SD of coefficients of covariates for the full model after running the elastic net model 10 times.**

|  | **s=lambda.min** | |
| --- | --- | --- |
| **Covariates** | **Mean** | **SD** |
| Center3 | -0.373 | 0.089 |
| Sex | -0.368 | 0.130 |
| Center1 | -0.286 | 0.101 |
| Isoleucyl-proline | -0.210 | 0.036 |
| DG(31:2) | -0.145 | 0.065 |
| N6,N6,N6-Trimethyl-L-lysine | -0.144 | 0.056 |
| Cysteinyl-Proline | -0.136 | 0.055 |
| Proline betaine | -0.108 | 0.030 |
| PC(O-18:0/22:6) | -0.099 | 0.046 |
| BMI | -0.099 | 0.010 |
| Acetylphosphate | -0.097 | 0.051 |
| Inositol-1,3,4,5-tetraphosphate | -0.091 | 0.031 |
| PC(O-16:0/18:3) | -0.078 | 0.020 |
| LysoPS(O-18:0/0:0) | -0.076 | 0.064 |
| LysoPC(20:5) | -0.075 | 0.041 |
| PC(18:0/18:2) | -0.074 | 0.034 |
| PC(O-18:0/22:4) | -0.071 | 0.034 |
| Tranexamic Acid | -0.069 | 0.038 |
| SM(d18:1/18:1) | -0.068 | 0.045 |
| PC(O-18:0/22:5) | -0.065 | 0.023 |
| Leucylproline | -0.060 | 0.041 |
| Ketoleucine | -0.060 | 0.073 |
| LysoPC(20:3) | -0.059 | 0.061 |
| 6-Deoxocastasterone | -0.053 | 0.061 |
| LysoPC(20:2) | -0.049 | 0.045 |
| Inosine | -0.042 | 0.036 |
| Lactic acid | -0.040 | 0.044 |
| PI(18:0/22:5) | -0.039 | 0.044 |
| SM(d18:2/24:1) | -0.039 | 0.041 |
| Dimethylglycine | -0.033 | 0.031 |
| Hippuric acid | -0.029 | 0.025 |
| PC(O-12:0/O-12:0) | -0.028 | 0.035 |
| PI(17:0/20:4) | -0.027 | 0.047 |
| PC(P-18:1/22:6) | -0.023 | 0.028 |
| PE(24:0/24:1) | -0.023 | 0.034 |
| 3-hydroxyoctanoyl carnitine | -0.022 | 0.036 |
| Hypoxanthine | -0.021 | 0.031 |
| DG(33:5) | -0.020 | 0.033 |
| 3-Methylglutarylcarnitine | -0.020 | 0.022 |
| Nα-Acetyl-L-arginine | -0.019 | 0.027 |
| PC(P-18:1/20:5) | -0.019 | 0.021 |
| PC(16:0/20:4) | -0.017 | 0.024 |
| Ecgonine | -0.017 | 0.036 |
| Sulfate | -0.017 | 0.021 |
| Cer(d16:2/22:1(2OH)) | -0.016 | 0.038 |
| N2-Acetyl-L-ornithine | -0.015 | 0.025 |
| PECer(d16:1/24:1) | -0.014 | 0.029 |
| Indoxylsulfuric acid | -0.013 | 0.017 |
| PC(O-22:2/22:3) | -0.013 | 0.029 |
| 2-Piperidinone | -0.011 | 0.024 |
| PE(16:0/22:6) | -0.011 | 0.019 |
| PE(O-16:0/18:3) | -0.011 | 0.014 |
| PC(O-16:0/20:5) | -0.011 | 0.014 |
| SM(d18:2/15:0) | -0.011 | 0.026 |
| 2-Methyl-1-Pyrroline | -0.009 | 0.013 |
| PE(P-18:1/22:6) | -0.009 | 0.018 |
| 13Z-Docosenamide | -0.008 | 0.015 |
| 6-Deoxodolichosterone | -0.005 | 0.015 |
| Nicotine glucuronide | -0.005 | 0.015 |
| Ectoine | -0.005 | 0.015 |
| Leucinic acid | -0.004 | 0.013 |
| PI(16:0/18:2) | -0.004 | 0.010 |
| Dihydroxybenzoic acid | -0.004 | 0.009 |
| SM(d18:1/15:0) | -0.004 | 0.013 |
| PC(16:0/20:5) | -0.003 | 0.010 |
| SM(d18:1/21:0) | -0.003 | 0.009 |
| DG(32:4) | -0.003 | 0.008 |
| L-Ornithine | -0.002 | 0.008 |
| Xanthine | -0.002 | 0.006 |
| L-Threonine | -0.001 | 0.003 |
| N-Acetylglucosamine 6-sulfate | -0.001 | 0.004 |
| SM(d18:0/18:0) | -0.001 | 0.003 |
| LysoPE(P-16:0/0:0) | -0.001 | 0.002 |
| Hydroxyhexanoycarnitine | 0.000 | 0.000 |
| PC(P-16:0/20:5) | 0.000 | 0.000 |
| SM(d18:0/16:0) | 0.000 | 0.000 |
| cholesterol sulfate | 0.000 | 0.000 |
| PS(O-18:0/18:2) | 0.000 | 0.001 |
| 3-Hydroxyisovalerylcarnitine | 0.000 | 0.002 |
| L-Arginine | 0.001 | 0.002 |
| PC(16:0/22:6) | 0.001 | 0.002 |
| PS(22:0/22:4) | 0.001 | 0.002 |
| LysoPC(18:1) | 0.001 | 0.002 |
| 2-Hexenoylcarnitine | 0.001 | 0.003 |
| LysoPE(22:6/0:0) | 0.001 | 0.004 |
| SM(d18:2/21:0) | 0.001 | 0.004 |
| PI(O-16:0/22:6) | 0.001 | 0.004 |
| SM(d18:1/19:0) | 0.002 | 0.005 |
| CerP(d18:1/14:0) | 0.002 | 0.005 |
| N1-Acetylspermidine | 0.003 | 0.008 |
| LysoPC(20:1) | 0.003 | 0.008 |
| SM(d18:2/14:0) | 0.005 | 0.011 |
| Acetylglycine | 0.005 | 0.015 |
| SM(d18:1/17:0) | 0.006 | 0.019 |
| PC(18:1/22:6) | 0.006 | 0.014 |
| LysoPE(18:1/0:0) | 0.007 | 0.014 |
| LysoPE(20:4/0:0) | 0.009 | 0.029 |
| LysoPE(20:2/0:0) | 0.009 | 0.028 |
| 1-Hydroxyvitamin D5 | 0.010 | 0.022 |
| Glycerophosphocholine | 0.010 | 0.023 |
| 3, 5-Tetradecadiencarnitine | 0.011 | 0.012 |
| L-Valine | 0.012 | 0.033 |
| PC(18:0/22:6) | 0.014 | 0.043 |
| LysoPE(22:1/0:0) | 0.017 | 0.030 |
| Diethanolamine | 0.017 | 0.032 |
| L-Tyrosine | 0.017 | 0.036 |
| Deoxycholic acid | 0.017 | 0.032 |
| LysoPC(24:0/0:0) | 0.017 | 0.025 |
| Sphinganine | 0.020 | 0.030 |
| 1-Methyladenosine | 0.022 | 0.035 |
| N-Oleoyl-L-Serine | 0.023 | 0.027 |
| 3-Deoxyvitamin D3 | 0.027 | 0.038 |
| Niacinamide | 0.028 | 0.026 |
| LysoPS(22:4/0:0) | 0.030 | 0.035 |
| PE(16:0/22:4) | 0.036 | 0.033 |
| PC(16:0/18:2) | 0.037 | 0.063 |
| PC(16:0/18:3) | 0.039 | 0.053 |
| PS(O-18:0/22:6) | 0.043 | 0.037 |
| L-α-Hydroxyisovaleric acid | 0.046 | 0.037 |
| Age | 0.051 | 0.003 |
| DL-Stearoylcarnitine | 0.053 | 0.060 |
| L-Homocitrulline | 0.053 | 0.028 |
| Pantothenic Acid | 0.057 | 0.058 |
| Alpha-D-Fucose | 0.086 | 0.067 |
| PS(O-18:0/20:4) | 0.100 | 0.036 |
| PECer(d15:1/22:0(2OH)) | 0.102 | 0.056 |
| 3-Dehydroteasterone | 0.120 | 0.073 |
| LysoPC(19:0/0:0) | 0.131 | 0.076 |
| Center2 | 0.137 | 0.116 |
| Dodecanoylcarnitine | 0.149 | 0.055 |
| Prolyl-Hydroxyproline | 0.166 | 0.079 |
| 5-Hydroxyectoine | 0.214 | 0.046 |
| Center4 | 0.424 | 0.100 |
| CTX-I | 1.307 | 0.127 |

SD, standard deviation; BMI, body mass index; CTX-I, C-terminal telopeptide of type I collagen.
